# A role for the gibberellin pathway in biochar-mediated growth promotion

**DOI:** 10.1038/s41598-018-23677-9

**Published:** 2018-03-29

**Authors:** Elizabeth French, Anjali S. Iyer-Pascuzzi

**Affiliations:** 1 Purdue University, Department of Botany and Plant Pathology, 915 W. State Street, West Lafayette, Indiana 47906 USA; 2 Purdue Center for Plant Biology, Purdue University, West Lafayette, IN USA

## Abstract

Biochar is a carbon negative soil amendment that can promote crop growth. However, the effects of biochar on different plant species and cultivars within a species are not well understood, nor is the underlying basis of biochar-mediated plant growth promotion. This knowledge is critical for optimal use of biochar and for breeding biochar-responsive plants. Here, we investigated the genotype-specific effects of biochar on two cultivars of *Solanum lycopersicum* (tomato), and two wild relatives of tomato, *Solanum pimpinellifolium*, and *Solanum pennelli*, in two types of biochar. Biochar promoted shoot growth in all genotypes independent of biochar type but had genotype-dependent effects on other plant traits. Germination tests, exogenous GA_4_ application and mutant analysis indicated a role for GA in biochar-mediated plant growth promotion. Together, our results suggest that biochar promotes growth partially through stimulation of the GA pathway.

## Introduction

Biochar is a carbon negative soil amendment produced from pyrolysis of organic material. Its potential for mitigating climate change and improving agricultural soils was recognized after the discovery of Terra Preta soil in the Amazon, where soils conditioned with black carbon additions thousands of years ago by native residents continue to be more fertile and carbon-rich than surrounding soils even today^1,2^. Today, soil amendment with biochar is being evaluated as a strategy for improving soil fertility, while simultaneously sequestering carbon and reducing greenhouse gas emissions^3^. Overall, modern biochars appear to promote plant growth^4^, though some studies have documented mixed or even negative effects of biochar^5–13^.

Research on how biochar promotes plant growth has focused on its positive impacts on soil characteristics, nutrient availability (reviewed in refs^4,14^) and the soil microbial community (reviewed in ref.^15^). Additionally, several groups have demonstrated that biochar is effective in controlling foliar and soil-borne pathogens with both biotrophic and necrotrophic lifestyles on multiple host crops (reviewed in refs^16,17^). However, others have shown a neutral or negative effect of biochar on disease progress^18–21^. Evidence indicates that differences in environment as well as biochar feedstock and production conditions each play a role in biochar’s effectiveness^4^. A recent meta-analysis of biochar studies showed that biochar’s effect on total plant biomass differs between annual and perennial crops^4^, suggesting that the effect of biochar on crop growth promotion may be dependent on crop species. Although the complexity of determinants underlying the agricultural outcomes of biochar amendment is not fully understood, further insight into the differences in biochar response both between and within species could improve biochar’s use in agriculture and lead to breeding for biochar responsiveness.

The bulk of biochar research on agricultural productivity has thus far focused on biochar’s effects on overall shoot growth and yield in major crops^4,22–29^. Fewer studies have examined the effects of biochar on particular growth traits that impact yield, such as germination, shoot or root architecture^30–34^. Understanding how specific growth traits are impacted by biochar addition will lead to improved uses for biochar, such as in germination potting mixes for use in greenhouse applications.

The applicability of such data is maximized by understanding the plant signaling pathways underlying the positive effects of biochar on plant growth. Microarray-based genome-wide transcriptional analysis in Arabidopsis^35^ demonstrated that biochar application increased transcription of auxin- and brassinosteroid- related genes with a concurrent decrease in defense-related genes, which the authors suggested indicated a tradeoff between growth and defense in biochar-grown plants. Further elucidation of the underlying basis for how biochar functions to promote growth and influence disease will lead to improved practices for biochar use and potentially new synergistic applications with other horticultural or agronomic practices.

In this study, our objectives were to understand the species-specific, within-species (e.g. cultivar), and trait-specific aspects of biochar-mediated plant growth promotion. We hypothesized that biochar would promote growth independent of crop species and plant trait. To test this, we first examined the effect of two different biochars on various growth traits of two cultivars of tomato (*Solanum lycopersicum*) and two species that are wild tomato relatives (*S. pimpinellifolium* and *S. pennellii*) in greenhouse conditions. Surprisingly, we found that although biochar promotes shoot growth independent of genotype, it has genotype-specific effects on other traits. Based on these results, we then aimed to understand the underlying basis for these phenotypes. The effects of biochar on plant growth suggested the involvement of the gibberellin (GA) pathway. We hypothesized that this pathway may play a role in biochar-mediated plant growth promotion. To further investigate this, we used hormone assays and mutant analysis. These experiments revealed a functional role for GA pathways in biochar-induced growth promotion.

## Results

### Biochar promotes shoot growth in a genotype-independent manner

In order to test the effects of biochar addition on different tomato genotypes, we performed a full factorial greenhouse experiment on two *S. lycopersicum* cultivars, *S. pimpinellifolium*, and *S. pennellii* with two different types of biochar at 4% weight weight^−1^ and a control 0% application rate. The two biochars significantly differed in their pH, organic matter content, and mineral composition (Table 1). We performed two trials and measured shoot growth traits after eight weeks of growth. The first trial was performed from December to late February and the second from late March to late May. For simplicity, the two *S. lycopersicum* cultivars and two wild species will be referred to as four genotypes. Biochar was a significant effect in the model for both shoot length and fresh weight (Table 2). Genotype was also significant for both shoot growth parameters, reflecting the different growth patterns between genotypes (Table 2). Interestingly, the p-value for the interaction between genotype and trial was 0.098 for shoot length and 0.06 for shoot fresh weight, suggesting that the trial may have impacted growth of some genotypes. This may have been due to environmental differences between the two trials, with the first trial conducted during the winter months with shorter days compared to the second trial in the spring. The biochar*genotype interaction was not significant for either model, indicating that the effects of biochar on shoot length and fresh weight are genotype-independent. Because the effects of biochar were independent of genotype, we used one genotype (H7996) to examine the effect of each biochar on leaf nutrient content (Table S1). Results showed very small to no differences in the levels of N and C between control and biochar treated H7996 plants, but decreased levels of potassium (K).

**Table 1:** Biochar Characteristics.

	Units	Premium	Ultra
Moisture	%	6.41^1^ (±0.275)a^2^	6.02 (±0.158)a
pH		10.22 (±0.012)a	10.09 (±0.020)b
LOI^3^	%	91.97 (±0.410)b	96.06 (±0.217)a
Org. matter	%	64.15 (±0.287)b	67.01 (±0.152)a
Nitrogen	**%**	0.19 (±0.027)a	0.33 (±0.067)a
Carbon	**%**	75.78 (±1.204)b	80.79 (±1.005)a
Aluminum	mg kg^−1^	24.99 (±0.330)a	15.92 (±0.111)b
Arsenic	mg kg^−1^	1.32 (±0.123)b	8.26 (±1.583)a
Boron	mg kg^−1^	9.71 (±0.114)b	11.57 (±0.342)a
Barium	mg kg^−1^	38.53 (±0.600)a	38.27 (±0.299)a
Calcium	mg kg^−1^	8,151.66 (±146.05)a	5,217.97 (±56.99)b
Cadmium	mg kg^−1^	0.14 (±0.004)a	0.03 (±0.004)b
Cobalt	mg kg^−1^	0.05 (±0.003)a	0.07 (±0.010)a
Chromium	mg kg^−1^	0.12 (±0.005)a	0.13 (±0.008)a
Copper	mg kg^−1^	0.00 (±0.000)b	1.06 (±0.228)a
Iron	mg kg^−1^	1.36 (±0.020)a	0.70 (±0.044)b
Potassium	mg kg^−1^	3,499.82 (±25.75)b	3,696.03 (±12.58)a
Magnesium	mg kg^−1^	695.78 (±12.68)a	704.82 (±18.29)a
Manganese	mg kg^−1^	350.32 (±5.791)a	220.29 (±2.114)b
Molybdenum	mg kg^−1^	0.02 (±0.001)a	0.02 (±0.005)a
Sodium	mg kg^−1^	1,028.96 (±8.359)b	1,112.95 (±8.587)a
Nickel	mg kg^−1^	0.26 (±0.009)a	0.18 (±0.016)b
Phosphorus	mg kg^−1^	544.85 (±9.443)a	356.24 (±5.590)b
Lead	mg kg^−1^	1.36 (±0.091)a	0.20 (±0.031)b
Sulfur	mg kg^−1^	631.44 (±11.69)a	82.83 (±1.555)b
Selenium	mg kg^−1^	0.25 (±0.043)a	0.24 (±0.051)a
Silicon	mg kg^−1^	110.64 (±3.215)a	47.51 (±1.335)b
Strontium	mg kg^−1^	28.27 (±0.397)a	24.66 (±0.407)b
Titanium	mg kg^−1^	0.19 (±0.003)a	0.08 (±0.007)b
Vanadium	mg kg^−1^	0.00 (±0.005)a	0.00 (±0.001)a
Zinc	mg kg^−1^	6.57 (±0.076)a	1.63 (±0.133)b

^1^Values indicate averages of three technical replicates ± (standard error).

^2^Differing letters indicate differences between biochars by t-test at p < 0.05.

^3^LOI = loss on ignition.

**Table 2:** General linear mixed model results of effects of biochar treatment, genotype, trial and their interactions on shoot weight and length.

	Effect	Num DF^1^	Den DF	F Value	Pr > F
Shoot Length (cm)	Genotype	3	88	177.78	**<0.0001**
	Biochar	2	88	6.83	**0.0017**
	Trial	1	88	0.15	0.7010
	Genotype*Biochar	6	88	0.64	0.6975
	Genotype*Trial	3	88	2.16	0.0984
	Biochar*Trial	2	88	0.73	0.4838
	Genotype*Biochar*Trial	6	88	0.47	0.8325
Shoot Fresh Weight (g)	Genotype	3	88	14.63	**<0.0001**
	Biochar	2	88	39.79	**<0.0001**
	Trial	1	88	1.21	0.2739
	Genotype*Biochar	6	88	0.43	0.8551
	Genotype*Trial	3	88	2.54	0.0617
	Biochar*Trial	2	88	0.85	0.4324
	Genotype*Biochar*Trial	6	88	0.83	0.5488

^1^Degrees of Freedom.

**Figure 1:**
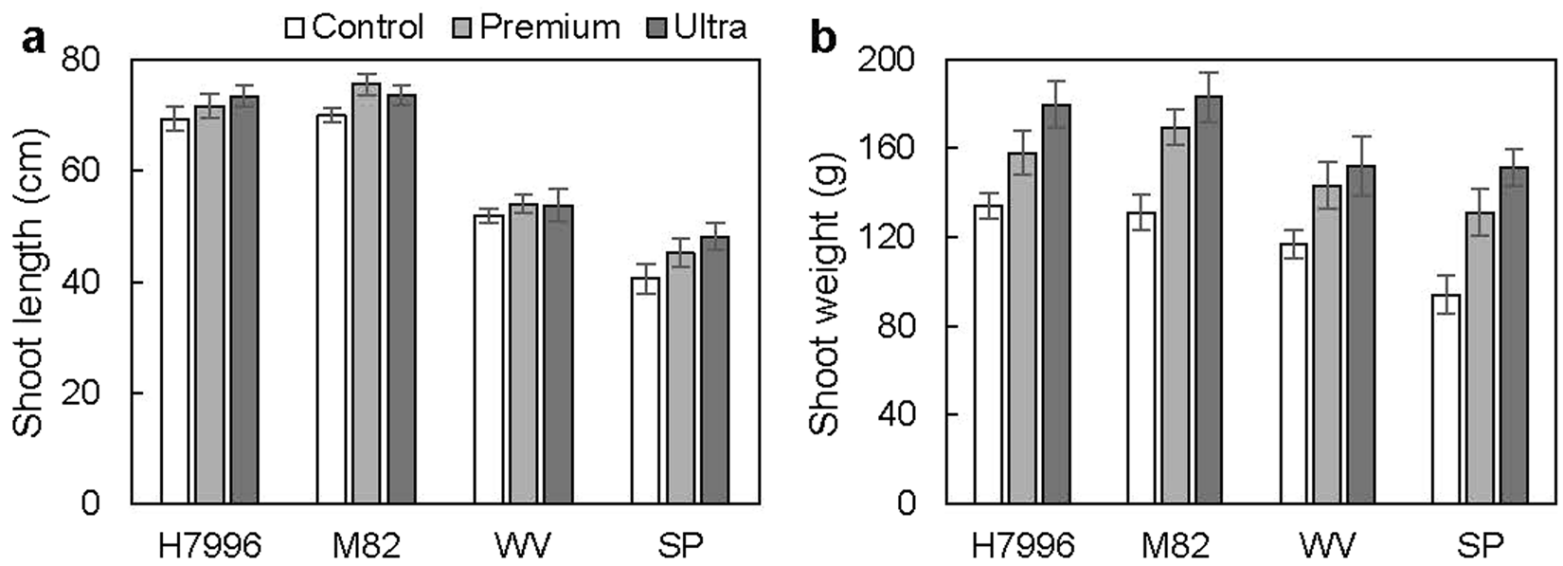
Biochar promotes shoot length and shoot weight in a genotype-independent manner. Graphs of (**a**) shoot length and (**b**) shoot fresh weight, showing the effects of biochar addition and genotype. ‘Control’ indicates no biochar addition, while ‘Premium’ and ‘Ultra’ represent 4% (w w^−1^) soil amendment with indicated biochar. Because a significant interaction between genotype and biochar addition was not observed (Table 2), here we compared the effect of different biochar types with the control. For this, data from different genotypes were pooled within each biochar and within the control. A post-hoc Tukey’s honestly significant differences test comparing biochar types and the control indicated significant differences between control and biochar amendments for both shoot length and weight. Abbreviations: H7996 – *Solanum lycopersicum* cv. Hawaii7996. M82 – *S. lycopersicum* cv. M82. WV – *S. pimpinellifolium* accession West Virginia700. SP – *S. pennellii* accession LA0716.

Overall, both types of biochar (Premium and Ultra) significantly promoted shoot weight and shoot length over the control plants (Table 2; Fig. 1). Premium biochar increased shoot weight by an average of 33.1% (±6.9%) and shoot length by 8.0% (±2.6%). Ultra biochar increased shoot weight by 50.9% (±11.6%) and shoot length by 9.2% (±3.1%). Ultra biochar promoted shoot weight, but not shoot length, significantly more than Premium biochar (Fig. 1).

### Biochar amendment affects germination and time to flowering in a genotype-specific manner

**Figure 2:**
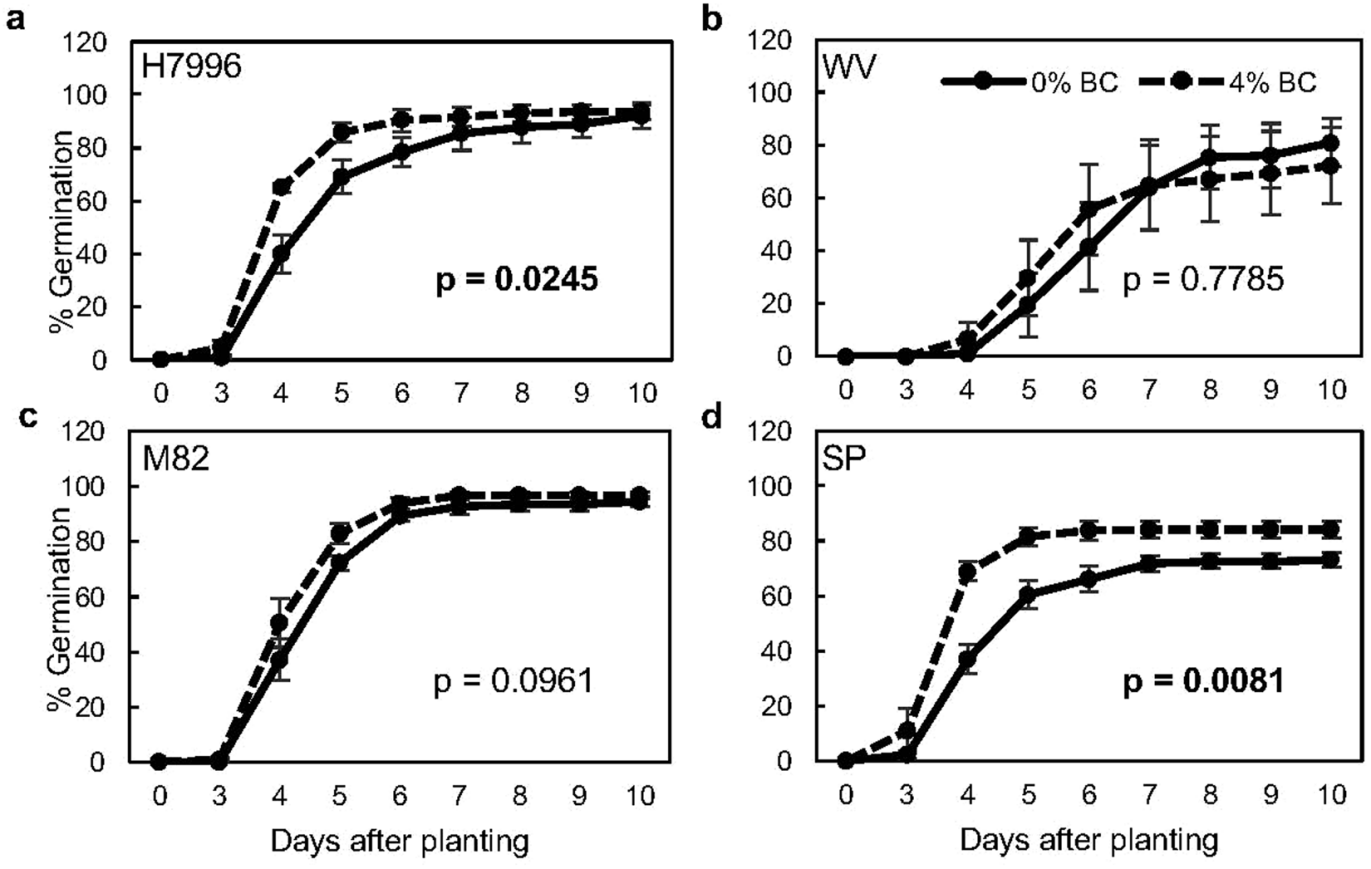
Premium biochar reduces time to germination and increases germination percentage in two tomato genotypes. (**a**–**d**) Percent germination over time in genotypes H7996, WV, M82, and SP, respectively, in 0 and 4% Premium biochar amended potting mix. Results in (**a**–**d**) are the averages of 54 seeds/treatment with four biological replicates. Error bars represent one standard error. P value represents mixed model ANOVA comparing Area Under the Germination Curve (AUGPC) values between biochar-treated and un-treated pots for each genotype. P value was considered significant at p < 0.05 (bold). Abbreviations: H7996 – *Solanum lycopersicum* cv. Hawaii7996. M82 – *S. lycopersicum* cv. M82. WV – *S. pimpinellifolium* accession West Virginia700. SP – *S. pennellii* accession LA0716.

**Figure 3:**
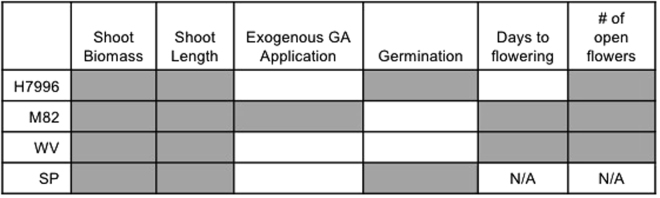
Summary of the effects of biochar on growth traits, separated by plant genotype. Gray boxes indicate that biochar had a positive effect on that trait for that genotype. White boxes indicate no effect. Flowering traits for SP are marked N/A because control SP plants had not flowered by the end of the experiment, so no statistical analysis could be performed.

In order to further investigate biochar’s role in other growth traits, we examined the effect of biochar on germination. Seeds from all four genotypes were planted with or without 4% Premium biochar and were measured for germination over a 10 day period. Area under the germination progress curves (AUGPC) for each of the four genotypes revealed that Premium biochar increased germination rate in H7996 and SP (Fig. 2a,d). Overall germination increased for SP by nearly 20% (Fig. 2d). AUGPC differences for germination in M82 between biochar-grown and control seeds were p < 0.1, suggesting a possibly similar, but weaker trend to H7996. These results suggest a species-specific effect of biochar on germination, as only 2 (*S. lycopersicum* and *S. pennellii*) out of 3 species showed a germination phenotype with biochar addition. Consistent with our observations that biochar affects GA-related traits, we observed both a decrease in days to flowering and an increase in the number of flowers at eight weeks in the second trial of the greenhouse experiment (Fig. S1). A summary of the effect of biochar in different *Solanum* species and within a given *Solanum* species is in Fig. 3.

### Biochar water extracts affect *S. pennellii* seedling growth traits

After observing the effect of biochar on growth under controlled growth conditions, we hypothesized that this phenotype was due to a direct effect of chemicals present in the biochar. In order to test this hypothesis, we made water extracts of both biochars and measured growth of *S. pennellii* (SP) on agar plates (Fig. S2). We chose SP because it is the most amenable to growth on agar plates and responded highly to biochar amendment. Seedlings treated with Ultra and Premium biochar extracts exhibited higher seedling weight and longer hypocotyls compared to the control (Fig. S2a,b). Root length was only affected by Premium water extracts compared to the control (Fig. S2c). These results suggest that biochar’s growth promoting effects in the greenhouse trials come at least in part from water-soluble compounds present in the biochar.

We hypothesized that Premium biochar may contain karrikins, germination promoting compounds found in smoke and other biochars^36^ that require the GA pathway to promote germination^37^. GC-MS analysis was performed on ethyl acetate extracts of ground Premium biochar to detect the presence of karrikins. However, no karrikins were detected in Premium biochar (Fig. S3).

### Biochar amendment increases response to exogenous GA in one tomato genotype

**Figure 4:**
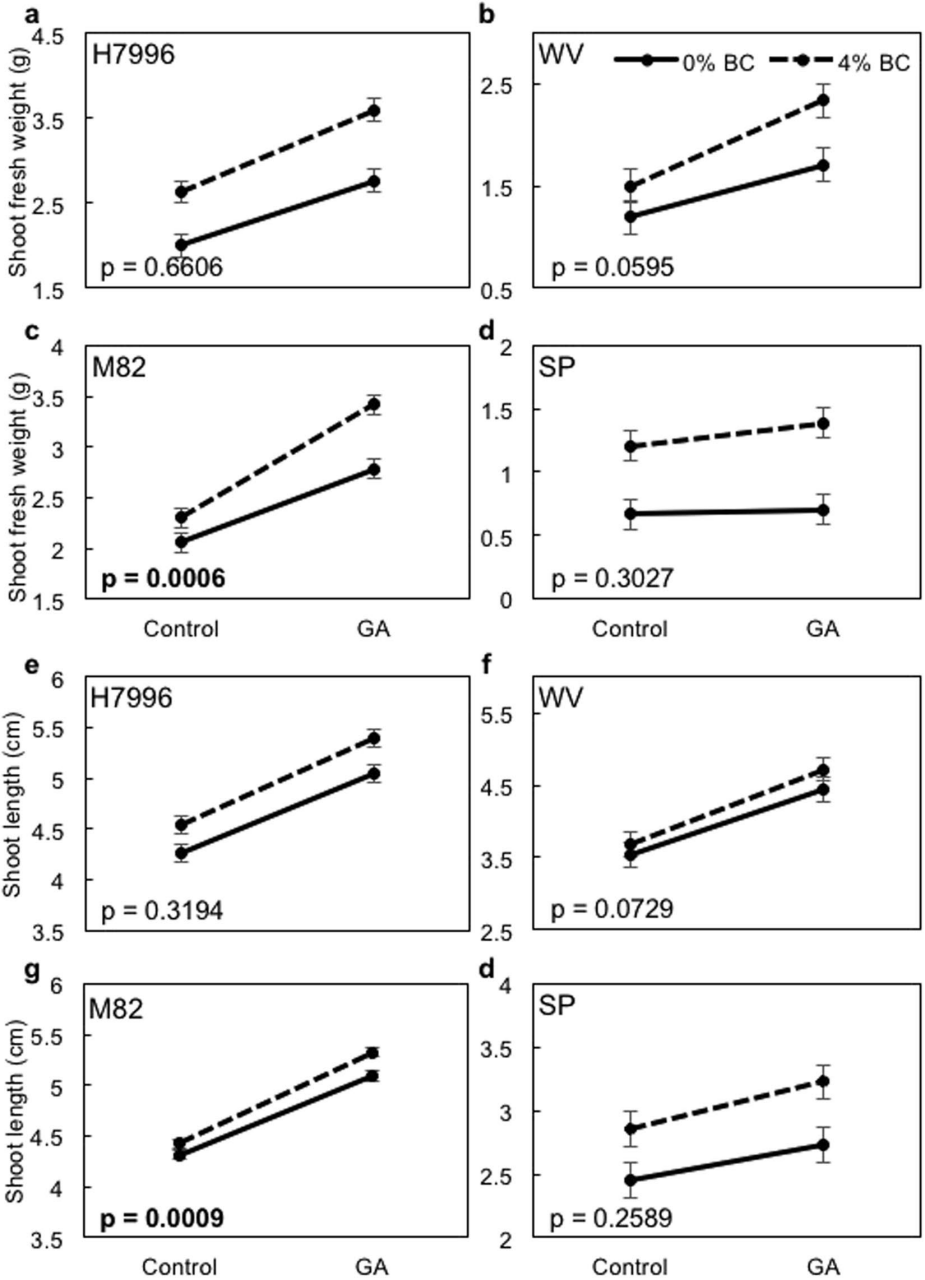
Exogenous gibberellin (GA_4_) application and Premium biochar (BC) amendment interact synergistically to increase the shoot biomass and length of M82 tomato plants. Least Square (LS) Mean interaction plots for each species show relationship between GA_4_ application and BC amendment for (**a**–**d**) shoot fresh weight and (**e**,**f**) shoot length. The experiment was repeated in four trials, and trial was included as a random factor in the model. Shoot weight and length values were square root transformed to meet homogeneity of variance and normality assumptions. Values represented in the figure are not transformed. P values represent significance of BC × GA_4_ interaction in the full model analysis. The interaction effect was considered statistically different at p < 0.05. Error bars represent one standard error. Abbreviations: H7996 – *Solanum lycopersicum* cv. Hawaii7996. M82 – *S. lycopersicum* cv. M82. WV – *S. pimpinellifolium* accession West Virginia700. SP – *S. pennellii* accession LA0716. Full model results are in Table S1.

Because the growth phenotypes observed above (shoot length, germination, flowering time) were reminiscent of growth mediated through the gibberellin (GA) pathway, we asked whether biochar promoted plant growth through the GA pathway. To examine this, we first tested whether exogenous GA treatment differentially affected plants grown with or without biochar. We examined the effects of exogenous GA_4_ treatment on shoot growth in all four tomato genotypes grown with or without Premium biochar (Fig. 4). Our results show a positive, interactive effect of biochar and GA_4_ treatment on both shoot fresh weight and shoot length in one genotype, M82, suggesting that biochar stimulates the GA pathway (Fig. 4c,g). This effect appears to be within-species specific as the interaction effect was observed in M82 and not H7996, both *S. lycopersicum* cultivars (Fig. 4a,c,e,g). Full model results are in Table S1. A summary of the species- and within-species specific effects of biochar can be found in Fig. 3.

### Biochar induced growth promotion requires an intact GA biosynthesis pathway in Arabidopsis thaliana

**Figure 5:**
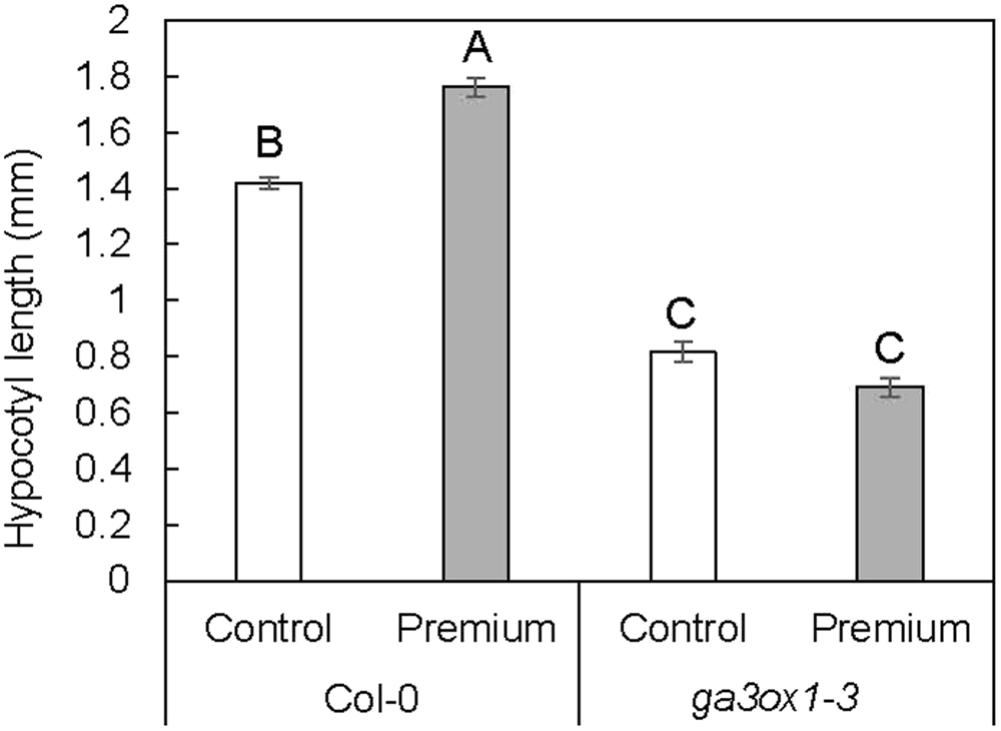
Premium biochar water extracts promote hypocotyl growth in WT Col-0, but not in *ga3ox1-3* mutant. Hypocotyl length of Col-0 or *ga3ox1-3* seedlings plated with Premium biochar water extract vs. sterile water treatment. Results represent the averages of three plates/treatment of 7–29 seeds/plate with two biological replicates performed. Square root transformed values were used for statistical analysis to meet homogeneity of variance assumption. Values represented in the figure are not transformed. Significant differences between all genotype and treatment combinations determined by Tukey’s honest significant differences at p < 0.05 indicated by differing letters. Error bars represent one standard error. Full model results can be found in Table S2.

In order to further evaluate the role of GA in biochar induced growth promotion, mutant analysis was performed. Because few tomato GA deficient mutants without severe growth defects exist, the Arabidopsis GA biosynthesis mutant *ga3ox1-3* was used. *ga3ox1-3* is defective in Gibberellin 3-oxidase 1 *(At1g15550)*, which is involved in the production of bioactive GA_4_ during the vegetative growth stage^38^. We hypothesized that if biochar promoted plant growth through the GA pathway, a mutant defective in GA biosynthesis would be less responsive to biochar. Growth assays on plates with biochar extracts showed that the GA pathway is required for a hypocotyl growth response to water extracts of biochar (Fig. 5, Table S2). Premium biochar extracts promoted hypocotyl growth in Col-0, the wild-type background for *ga3ox1-3* (Fig. 5). However, no significant difference was found in hypocotyl growth between control and Premium biochar extract-treated *ga3ox1-3* seedlings (Fig. 5). This result suggests that GA is at least partially responsible for biochar growth promotion in Arabidopsis.

## Discussion

Here we provide evidence that biochar-mediated growth promotion acts in part through the GA pathway. Further, we show that while the shoot length and fresh weight growth promotion effect of biochar is genotype-independent, the effect of biochar on other traits such as germination, depends on genotype. We demonstrated that biochar and exogenous GA application acted synergistically to affect tomato shoot growth, but only in cultivar M82. Our study highlights the complexity of biochar-plant interactions and helps explain some of the apparent contradictions in the biochar literature. Differences in growth or disease outcomes are commonly reported for different types and rates of biochar^13,18,24,31–33,39–43^. Additionally, previous studies have observed the full range of germination responses to biochar, from an inhibitory to a stimulatory effect^31,32,44–47^. One reason for these discrepancies could be due to the differential effects of biochar on different traits and genotypes. We showed that while biochar affected overall shoot growth across all genotypes, germination was only positively affected in two of the studied genotypes.

The trait-dependent effects of biochar have not been well characterized^31,32,42,48^. Here we show that, depending on the genotype, biochar impacts some growth traits but not others (germination or response to GA application, for example). Solaiman *et al*. (2012) tested the effects of different levels of five different biochars on three plant species and observed differing effects dependent on trait observed, plant species, biochar amendment level, and biochar type. For example, in wheat, biochar amendment increased germination and seedling growth at low amendment levels, but had negative effects at higher concentrations^31^. For mung bean and clover, biochar amendment had a negative effect on germination regardless of amendment level. Providing further evidence that biochar affects plant growth in a trait-specific manner, biochar did have positive effects on mung bean and clover growth at low concentrations, despite its negative effect on germination in these species^31^. While it is still unclear why the effects of biochar depend on the trait, species and cultivar examined, our discovery of within-species (e.g. cultivar) variation opens the door to the potential for breeding for a positive biochar response.

To test the hypothesis that GA is involved in biochar-mediated plant growth promotion, we examined biochar response traits related to GA and performed mutant analysis. Exogenous GA_4_ application revealed a positive, interactive effect of Premium biochar and GA application on shoot growth in M82, indicating that biochar stimulates the GA pathway. Similarly, Premium biochar promoted germination in H7996 and SP with the largest effect occurring in SP with approximately a 20% increase in percent germination over the control. Our experiments with the *ga3ox1-3* Arabidopsis mutant for GA biosynthesis supported the involvement of the GA pathway in biochar-mediated plant growth promotion. In another study, Soybean plants grown in 5% biochar exhibited upregulated transcription of β-1,4-glucanase, involved in cell wall expansion, which is a hallmark of GA-mediated growth^21^. Conversely, Viger *et al*. (2014) found evidence for upregulation of auxin and brassinosteroid pathways in Arabidopsis plants grown in biochar, though this may be due to differences in species response or the type of biochar used^35^. Our study supports evidence for a model by which biochar promotes tomato growth partly through stimulation of germination and growth through the GA pathway. Future work should focus on measuring hormone levels and transport in biochar-treated plants to further understand biochar’s influence on hormone pathways.

Biochar’s positive effects on shoot growth and germination, even under well-watered and fertilized conditions, suggest a direct effect of compounds present in the biochar on the plant. Our results showing that water-extracts of biochar promoted SP seedling growth confirmed this hypothesis. Though we were unable to identify karrikins in the biochar used here, recent studies have demonstrated that biochars contain bioactive compounds, including karrikins and humic substances products (HSP) which have been shown to have hormone-like effects on plant growth, including GA-like responses^36,49–51^. Alternatively, biochar’s effects on plant growth and defense may occur indirectly through its impacts on the soil microbial community. Biochar has been shown to shift root-associated communities toward microbes with plant-growth promoting or defense-promoting capabilities^13,52–54^. These altered communities may, in turn, impact plant growth and defense.

Our data supports a model in which biochar application stimulates the GA pathway in tomato and Arabidopsis. These data may lead to new potential applications for biochar, such as enhancing current horticultural practices like spraying exogenous GA on grapes for larger fruit production. We have also shown that while biochar generally promotes tomato growth under controlled greenhouse conditions, it affects specific traits such as germination in a genotype- and trait-specific manner. Future studies are needed to better understand how biochar affects plant hormone pathways and to examine how genetic differences influence plant responses to biochar in order to use biochar more effectively.

## Methods

### Biochar and Leaf Tissue Analysis

Premium and Ultra biochars were obtained from Black Owl Biochar in Washington state, USA (http://www.biocharsupreme.com/). Both were produced from sustainably managed Douglas fir under different commercial production conditions. Biochar and leaf tissue were chemically analyzed by the Cornell Nutrient Analysis Laboratory following methods from the Soil Survey Laboratory Methods Manual created by the National Soil Survey Center (Soil Survey Staff, 2014). Five leaves of H7996 from replicate 1 were pooled for leaf tissue analysis.

### Seed sterilization

For all experiments, tomato seeds were sterilized by shaking in 10% bleach for 10 minutes, and then rinsed six times in sterile double distilled water (ddH_2_O). Seeds were then left in sterile water overnight in a 4 °C refrigerator to imbibe. *Arabidopsis thaliana* seeds were prepared by allowing to stratify in sterile water in a 4 °C refrigerator for five days. Seeds were then sterilized by shaking in 1 mL 50% bleach and 1 µL Tween for five minutes and then rinsing in sterile ddH_2_O five times.

### Tomato Growth in Biochar

A full-factorial greenhouse experiment was designed to test the effects of biochar addition on the growth response of two *Solanum lycopersicum* (tomato) cultivars and two wild tomato species, *S. pimpinellifolium* and *S. pennellii*. The tomato cultivars used were Hawaii7996 (H7996), known for its disease resistance^55^ and M82, an inbred processing tomato cultivar^56^. The *Solanum pimpinellifolium* accession used was West Virginia700 (WV), known for its susceptibility to the bacterial pathogen *Ralstonia solanacearum*^57^. The *Solanum pennellii* (SP) accession was LA0716, which was recently sequenced^58^. For simplicity, the two tomato cultivars and two wild species will be referred to as four genotypes. Two types of biochar made from the same feedstock under different production conditions were used: Premium and Ultra. A custom soilless potting mix was made that consisted of a 1:1 (volume volume^−1^) ratio of peat to Turface MVP (Turface Athletics, Buffalo Grove, IL, USA). Biochars were amended into the potting mix at a rate of 4% biochar (weight weight^−1^) and mixed by hand. All four genotypes were also grown in control pots not amended with biochar.

Sterile tomato seeds were planted into classic 300 size pots (about 2.5 L) (Nursery Supplies, Inc., Chambersburg, Pennsylvania, USA) and were grown in a light and temperature-controlled greenhouse (temperature setting 75–84 °F) that was regularly maintained for pests. Lights operated on a 16 hour on, 8 hour off long day cycle. Pots were watered two to five minutes, one to three times per day by drip irrigation to maintain adequate water status and fertilized with a solution of Peter’s Excel 15-5-15 NPK Cal-Mag Special (Hummert’s International, Earth City, Missouri, USA) at 80 ppm nitrogen (N) with every watering after plants reached first true leaf stage. Pots were organized into five randomized, complete blocks for statistical analysis. Each biochar treatment (0 or 4%) and tomato genotype combination had five replicates, and the full experiment was repeated in two independent trials. The first trial was from Dec 2014–Feb 2015 and the second from March–May 2015. In each trial, plants were harvested 8 weeks after planting and measured for shoot length and fresh weight. Days to flowering and number of open flowers were counted for the second trial only. Days to flowering was counted as number of days from germination to first open flowers.

Results were analyzed by three-way ANOVA with a general linear mixed model (PROC GLIMMIX) in SAS 9.4. Biochar treatment, genotype, and trial were included as fixed effects with all possible interactions between the three effects, and block was included as a random effect. Post-hoc tests were performed with Tukey’s honest significant differences test. No transformations were necessary to meet the homogeneity of variance and normality assumptions.

### Germination in potting mix

In order to determine the effect of Premium biochar amendment on germination, individual sterile seeds of each of the four genotypes (H7996, M82, WV, and SP) were planted into pots in 36 pot flats containing either 1:1 peat/turface potting mix or 1:1 peat/turface amended with 4% Premium biochar by weight. Fifty-four seeds per treatment and genotype were planted. Days to germination was defined as the number of days to cotyledon expansion. Germination was measured once per day for 10 days. The germination experiment was fully replicated in four trials. Percent germination was calculated as follows:

$${Percent}\,{germination}=\,\frac{{Number}\,{of}\,{germinated}\,{seeds}}{{Total}\,{number}\,{of}\,{planted}\,{seeds}}\times 100.$$

Area under the germination progress curve (AUGPC) was calculated by the trapezoidal integration method (Campbell and Madden, 1990). Statistical analysis was performed in JMP12 to compare AUGPC values between Premium biochar-amended and un-amended pots within each species with a linear mixed model with biochar treatment as a fixed effect and trial as a random effect. The effect of biochar treatment was considered significantly different at p < 0.05.

### Exogenous GA spray

To determine the effect of bioactive gibberellin (GA_4_) treatment on biochar-treated vs. untreated tomato plants, approximately 50 plants of each of the four genotypes were grown in +/− biochar potting mix in 36 pot flats. When plants were two weeks old, they were divided into two sets (between 12–27 individuals, depending on germination rates). Once per day for five days, one set was sprayed with 7.5 mg ml^−1^ GA_4_, while the other set was sprayed with water. Shoot length measurements were taken at the beginning (Day 1) and end of the experiment (Day 8), and shoot weights were also taken at the end (eight days after initial spray). The entire experiment was repeated four times. Shoot weight and shoot length after eight days were used for statistical analysis. Statistical analysis was performed using PROC GLIMMIX in SAS 9.4. Fixed effects included in the model were biochar treatment and GA_4_ treatment with their interaction, and trial was included as a random factor. Data were examined for homogeneity of variance and normality. Shoot weight and length values were square root transformed to meet the homogeneity of variance assumption. Differences in the biochar*GA_4_ interaction model effect were considered significant at p < 0.05.

### Arabidopsis *ga3ox1-3* mutant analysis

The *ga3ox1-3* mutant was used for analysis^38^. This mutant is defective in Gibberellin 3-Oxidase 1 (*At1g15550*), which catalyzes the production of bioactive gibberellin GA_4_ during the vegetative growth stage^38^. *ga3ox1-3* was obtained from the Arabidopsis Biological Resource Center (CS6943) and the homozygous mutant was confirmed using TDNA insertion PCR using primers from Mitchum *et al*. 2006.

Water extracts of Premium biochar were made by stirring 50 g of biochar in 1 L of ddH_2_O overnight at room temperature. After stirring, extract was filtered first using vacuum filtration with Whatman 42 filter paper to remove large particles, and then filter-sterilized with a 0.22 µM filter. Treated 1% agar plates were prepared by applying 2 mL of sterile ddH_2_O (control) or sterile biochar filtrate to the plate surface and allowing the liquid to sink into the plate. For Arabidopsis mutant growth assays on plates, approximately 30 sterile seeds each of WT (Col-0) and *ga3ox1-3* were plated onto treated 1% agar plates in a single row and placed upright in a growth chamber set to 24 °C, 16-hour day and 8-hour night cycle and average of 80 μmol m^−2^ s^−1^ light. After eight days, plates were scanned and measured in ImageJ for hypocotyl length. The entire experiment was replicated twice. Statistical analysis was performed using PROC GLIMMIX in SAS 9.4. Fixed effects included in the model were biochar treatment, genotype, and trial with their interactions, and plate was included as a random factor. Hypocotyl length values were square root transformed to meet homogeneity of variance and normality assumptions.

## Electronic supplementary material

All Supplementary Data
