# Supplementary material for: A role for the gibberellin pathway in biochar-mediated growth promotion
Source: Sci Rep. 2018 Mar 29;8:5389. doi: 10.1038/s41598-018-23677-9 (PMC5876386; doi:10.1038/s41598-018-23677-9)
Supplement: Supplementary file 1 — All Supplementary Data [file 41598_2018_23677_MOESM1_ESM.pdf]

**Article title:** A role for the gibberellin pathway in biochar-mediated growth promotion.
**Journal name:** Scientific Reports
**Authors:** Elizabeth A. French and Anjali S. Iyer-Pascuzzi
**Corresponding author affiliation:** Purdue University, Department of Botany and Plant Pathology and Purdue Center for Plant Biology, West Lafayette, IN, USA
**Corresponding author email:** asi2@purdue.edu

**SUPPORTING INFORMATION**

**Fig S1** Premium biochar (BC) reduces days to flowering and increases number of flowers

**Fig S2** Biochar water extracts positively affect seedling growth in *Solanum pennellii*

**Fig S3** Gas Chromatography-Mass Spectrometry (GC/MS) of ethyl acetate extracts of Premium biochar (BC)

**Table S1** Generalized linear models of the effects of Premium biochar treatment (BC) and gibberellin treatment ($GA_4$) on shoot weight and length, separated by genotype

**Table S2** Generalized linear model of the effects of Arabidopsis genotype and Premium biochar (BC) extract treatment on hypocotyl length

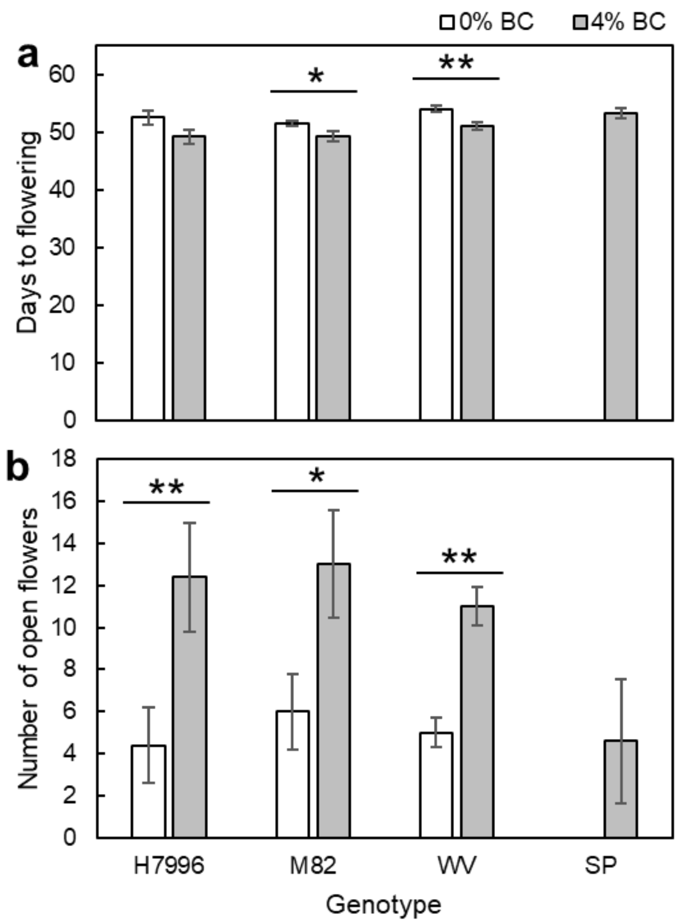


**Fig. S1** Premium biochar (BC) reduces days to flowering and increases number of flowers. a) Average number of days to flowering in all 4 genotypes grown in 0% BC or 4% Premium BC. b) Average number of open flowers in all 4 genotypes grown in 0% or 4% Premium BC. Asterisks indicate significance by t-test at the following levels: * $p < 0.1$, ** $p < 0.05$. Error bars represent one standard error. No statistical analysis performed on SP as no control plants had flowered by the end of the experiment (56 days after planting). Abbreviations: H7996 – *Solanum lycopersicum* cv. Hawaii7996. M82 – *S. lycopersicum* cv. M82. WV – *S. pimpinellifolium* accession West Virginia700. SP – *S. pennellii* accession LA0716.

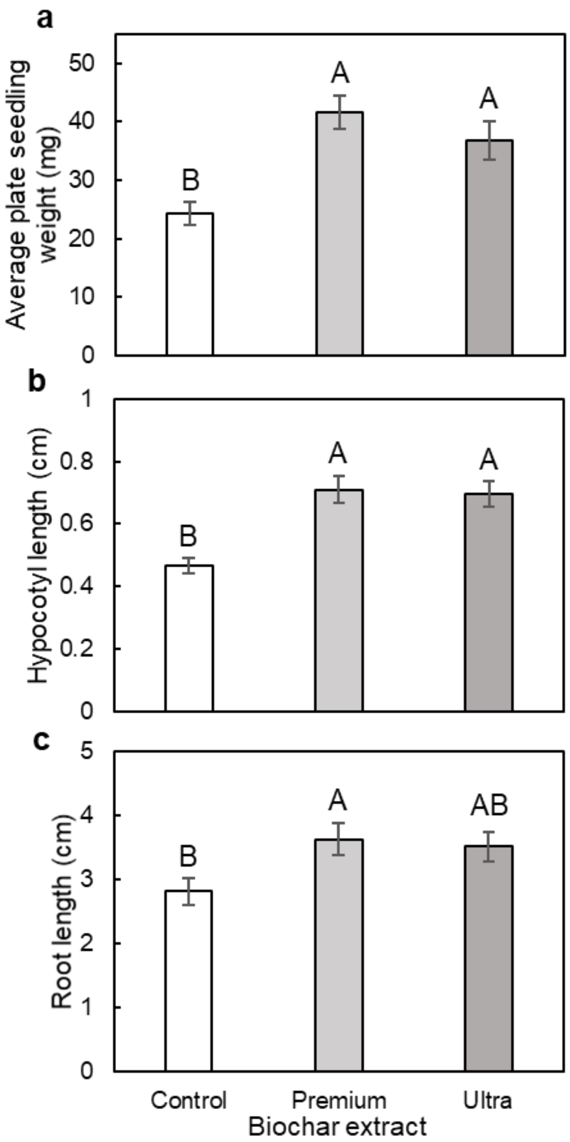


**Fig. S2** Biochar water extracts positively affect seedling growth in *Solanum pennellii*. a) average weight of pooled seedlings from each plate in milligrams (mg) b) average hypocotyl length in centimeters (cm) c) average root length in cm and of *S. pennellii* seeds plated with water (Control) or water extracts of the two biochars. Results represent the averages of five plates/treatment of 10 seeds/plate. Data displayed from first biological replicate. Experiment was performed four times with similar results. Significant differences between biochar treatments at $p < 0.05$ as determined by Tukey's honest significant differences indicated by differing letters for a-c. Hypocotyl length values were square root transformed to meet homogeneity of variance assumption. Values displayed are untransformed. Error bars represent one standard error.

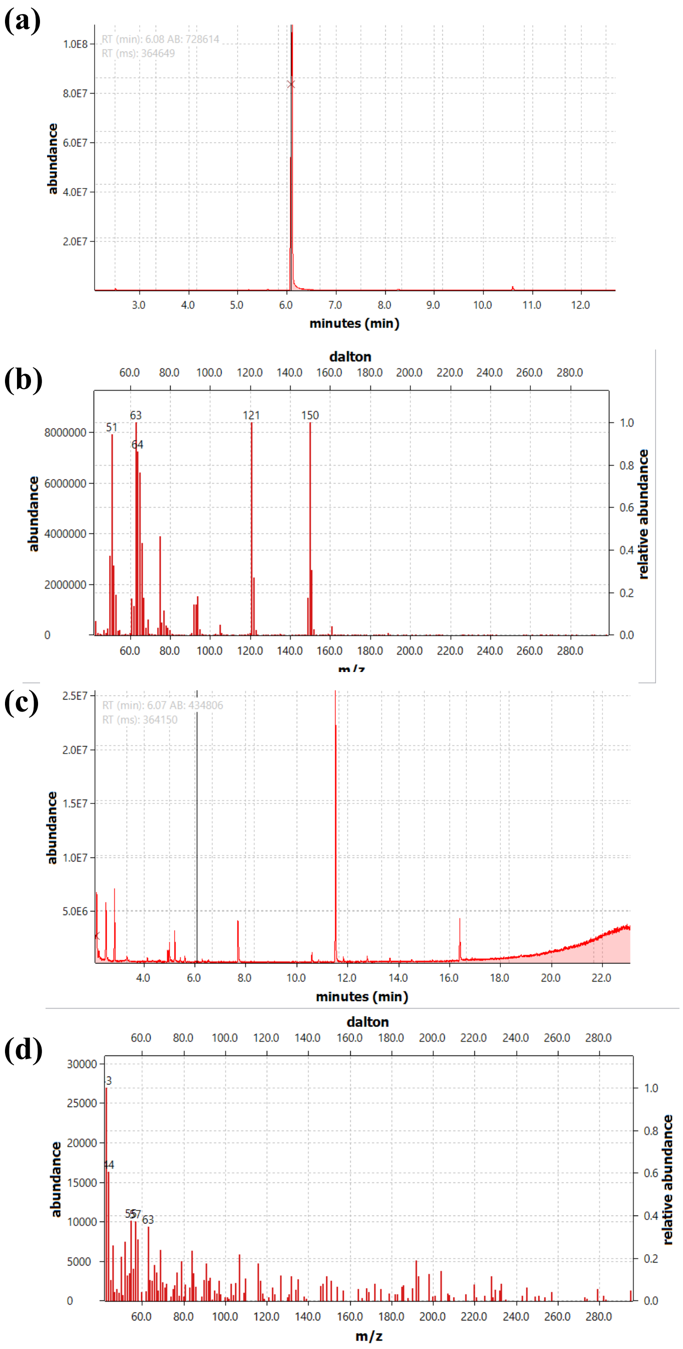


**Figure S3:** Gas Chromatography-Mass Spectrometry (GC/MS) of ethyl acetate extracts of Premium biochar (BC). a) Chromatogram of 1 mM karrikin 1 (KAR1) in ethyl acetate. Retention time ~6 min. B) Mass spectrum of KAR1 at 6.08 min retention time (marked as X in a). C) Chromatogram of ethyl acetate Premium BC extract. D) Mass spectrum at 6.09 min retention time.

**Supplementary Materials and Methods**

*Solanum pennellii seedling growth on plates.*

Control and Premium biochar extract treatments were prepared as for the Arabidopsis *ga3ox1-3* assays. For seedling growth experiments, 10 sterile *S. pennellii* seeds were plated onto treated plates in a single row and placed upright in a growth chamber set to 24 °C, 16-hour day and 8-hour night cycle and an average of 80 µmol $m^{-2}$ $s^{-1}$ light. After six days, plates were scanned and measured in ImageJ for root length and hypocotyl length. After scanning, seedlings from each plate were pooled and weighed together. Statistical analysis was performed in JMP12. Statistical differences were tested by ANOVA with treatment as a fixed effect and plate as a random effect, followed by a post-hoc Tukey test with a significance cut-off of $p < 0.05$. No transformations were necessary to meet the homogeneity of variance and normality assumptions. The experiment was repeated four times with similar results.

*KAR1 extraction and GC-MS analysis*

Premium biochar extraction was performed by first grinding 25 g biochar in a mortar and pestle. After grinding, biochar was stirred at room temperature with 100 ml ethyl acetate for two hours. Then biochar extract was filtered by vacuum filtration with Whatman 42 filter paper. Filtered extracts were then submitted along with 1 mM KAR1 standard to the Purdue Campus-Wide Mass Spectrometry Center. 1 µl samples were analyzed on an Agilent 5975C gas chromatograph-mass spectrometer (GC-MS). Sample separation was done on a DB-5 column 30 m in length, with a 0.25 mm inner diameter and 0.25 µm film thickness. The injection temperature was set to 250 °C. The temperature separation program for the GC was as

follows: 100 °C for 0.1 min, ramped at 10 °C to 320 °C. Mass spectra were scanned in the range of 42-300 amu. Chromatograms were visualized using OpenChrom 1.1.0[1].

## Supplementary References

1. Wenig, P. & Odermatt, J. OpenChrom: a cross-platform open source software for the mass spectrometric analysis of chromatographic data. *BMC Bioinformatics* **11,** 405 (2010)

**Table S1** Generalized linear models of the effects of Premium biochar treatment (BC) and gibberellin treatment ($GA_4$) on shoot weight and length, separated by genotype

| Type III Tests of Fixed Effects | | | | | | |
|---|---|---|---|---|---|---|
| **Genotype** | Response Variable | Effect | Num DF[1] | Den DF | F Value | Pr > F |
| **H7996** | Shoot Fresh Weight (g)[2] | BC | 1 | 372 | 118.66 | **<.0001** |
| | | GA | 1 | 372 | 135.26 | **<.0001** |
| | | BC*GA | 1 | 372 | 0.19 | 0.6606 |
| | Shoot Length (cm)[3] | BC | 1 | 372 | 118.50 | **<.0001** |
| | | GA | 1 | 372 | 761.81 | **<.0001** |
| | | BC*GA | 1 | 372 | 0.99 | 0.3194 |
| **M82** | Shoot Fresh Weight (g) | BC | 1 | 384 | 80.21 | **<.0001** |
| | | GA | 1 | 384 | 327.67 | **<.0001** |
| | | BC*GA | 1 | 384 | 11.94 | **0.0006** |
| | Shoot Length (cm) | BC | 1 | 384 | 73.37 | **<.0001** |
| | | GA | 1 | 384 | 1782.15 | **<.0001** |
| | | BC*GA | 1 | 384 | 11.26 | **0.0009** |
| **WV** | Shoot Fresh Weight (g) | BC | 1 | 305 | 59.30 | **<.0001** |
| | | GA | 1 | 305 | 145.68 | **<.0001** |
| | | BC*GA | 1 | 305 | 3.58 | 0.0595 |
| | Shoot Length (cm) | BC | 1 | 305 | 36.15 | **<.0001** |
| | | GA | 1 | 305 | 938.24 | **<.0001** |
| | | BC*GA | 1 | 305 | 3.24 | 0.0729 |
| **SP** | Shoot Fresh Weight (g) | BC | 1 | 266 | 140.70 | **<.0001** |
| | | GA | 1 | 266 | 2.42 | 0.1211 |
| | | BC*GA | 1 | 266 | 1.07 | 0.3027 |
| | Shoot Length (cm) | BC | 1 | 265 | 139.82 | **<.0001** |
| | | GA | 1 | 265 | 75.41 | **<.0001** |
| | | BC*GA | 1 | 265 | 1.28 | 0.2589 |

[1]Degrees of Freedom
[2]Shoot weight - square root transformed for analysis.
[3]Shoot length - square root transformed for analysis.

**Table S2** Generalized linear model of the effects of Arabidopsis genotype and Premium biochar (BC) extract treatment on hypocotyl length

| Type III Tests of Fixed Effects | | | | | |
|---|---|---|---|---|---|
| **Response Variable** | Effect | Num DF[1] | Den DF | F Value | Pr > F |
| **Hypocotyl length (mm)** [2] | Genotype | 1 | 479 | 544.65 | <.0001 |
| | BC | 1 | 479 | 9.14 | 0.0026 |
| | Genotype*BC | 1 | 479 | 25.94 | <.0001 |
| | Trial | 1 | 479 | 82.76 | <.0001 |
| | Genotype*Trial | 1 | 479 | 75.36 | <.0001 |
| | BC*Trial | 1 | 479 | 0.01 | 0.9038 |
| | Genotype*BC*Trial | 1 | 479 | 0.02 | 0.8818 |

[1]Degrees of Freedom

[2]Hypocotyl length – square root transformed for analysis
